# A supramodal and conceptual representation of subsecond time revealed with perceptual learning of temporal interval discrimination

**DOI:** 10.1038/s41598-022-14698-6

**Published:** 2022-06-23

**Authors:** Ying-Zi Xiong, Shu-Chen Guan, Cong Yu

**Affiliations:** School of Psychological and Cognitive Sciences, IDG/McGovern Institute for Brain Research, and Peking-Tsinghua Center for Life Sciences, Peking University, Beijing, China

**Keywords:** Human behaviour, Sensory processing

## Abstract

Subsecond time perception has been frequently attributed to modality-specific timing mechanisms that would predict no cross-modal transfer of temporal perceptual learning. In fact, perceptual learning of temporal interval discrimination (TID) reportedly shows either no cross-modal transfer, or asymmetric transfer from audition to vision, but not vice versa. However, here we demonstrate complete cross-modal transfer of auditory and visual TID learning using a double training paradigm. Specifically, visual TID learning transfers to and optimizes auditory TID when the participants also receive exposure to the auditory temporal interval by practicing a functionally orthogonal near-threshold tone frequency discrimination task at the same trained interval. Auditory TID learning also transfers to and optimizes visual TID with additional practice of an orthogonal near-threshold visual contrast discrimination task at the same trained interval. Practicing these functionally orthogonal tasks per se has no impact on TID thresholds. We interpret the transfer results as indications of a supramodal representation of subsecond time. Moreover, because TID learning shows complete transfer between modalities with vastly different temporal precisions, the sub-second time presentation must be conceptual. Double training may refine this supramodal and conceptual subsecond time representation and connect it to a new sense to improve time perception.

## Introduction

Understanding temporally dynamic events such as speech and music requires accurate perception of durations and intervals on a scale of subsecond. One long-standing debate regarding subsecond time perception is whether it is based on a dedicated central clock, which acts like a pacemaker-accumulator to keep track of the time^1,2^, or is intrinsic properties of neural dynamics that distribute over many sensory modalities and brain areas^3,4^. There is a large body of literatures on this topic^3,5^, but here we only focus on a subtopic, i.e., whether subsecond timing is modality specific. Modality specific time perception would more likely rely on distributed mechanisms, as evidenced in numerous studies^3,5^, including adaptation^6,7^ and perceptual learning^8–10^ studies. Otherwise, modality unspecific time perception would be more consistent with a dedicated central clock^11–14^, although a central clock could coexist and collaborate with distributed mechanisms for time perception^11,14–16^. Further, we only take on perceptual learning evidence that in general supports modality-specific subsecond time perception.

**Figure 1:**
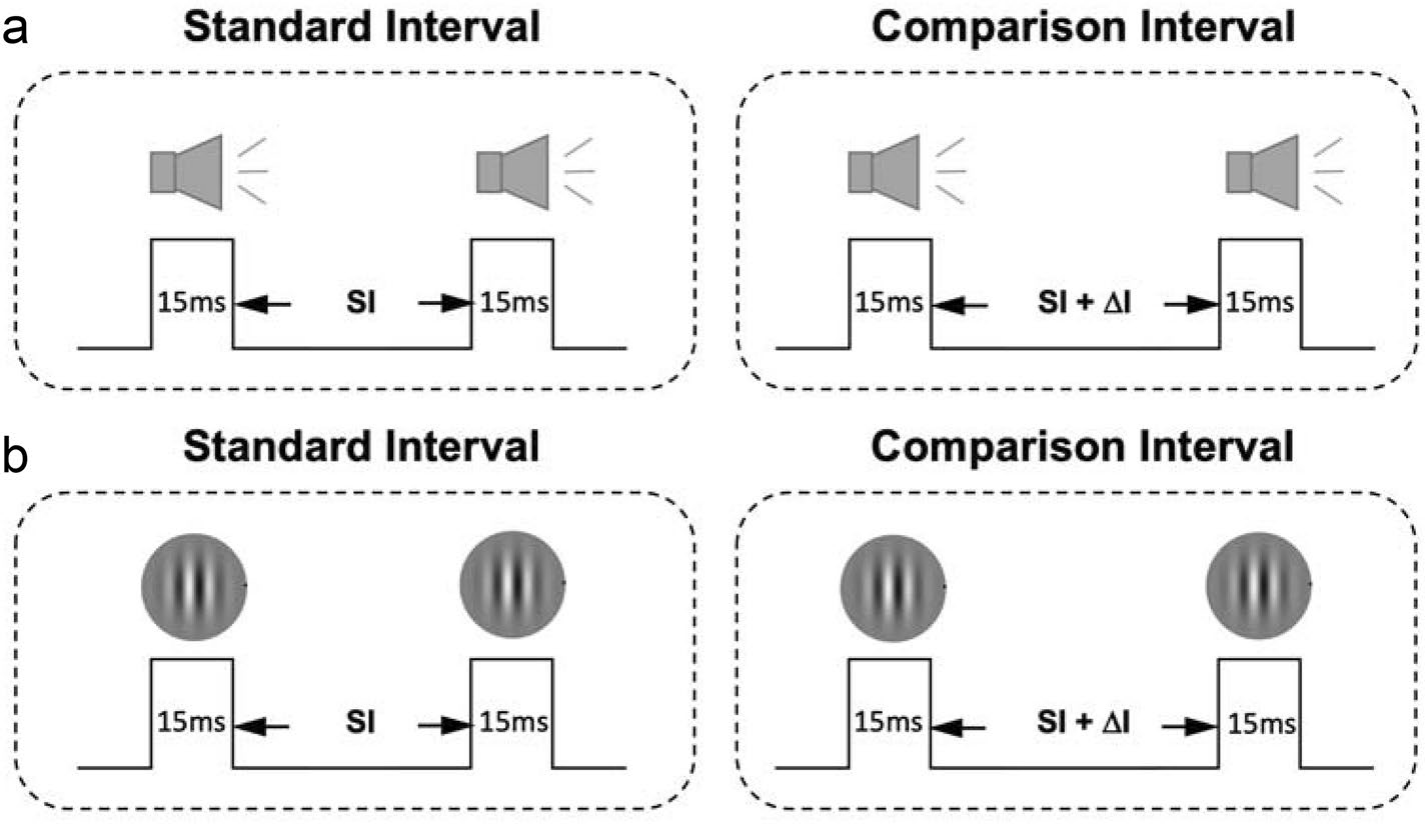
Illustrations of auditory and visual TID trials. (**a**) An auditory TID trial. The standard stimuli were two 15-ms tone pips separated by a 100 ms interval, and the comparison stimuli were the same two tone pips separated by a 100 + Δt ms interval. In a given trial, the standard and comparison stimuli were presented in random order with a 900 ms time gap. (**b**) A visual TID trial. The same as the auditory TID trial except that the tone-pips were replaced with Gabor patches.

Subsecond time perception can be improved through perceptual learning^17,18^. Because time intervals can be defined by visual, auditory, or other sensory stimuli, it is natural to assume that there is a supramodal representation of subsecond time in the brain, and that temporal perceptual learning from one modality should be able to transfer to another modality. However, this intuition is not supported by existing perceptual learning evidence. For example, an earlier study reported that perceptual learning of temporal interval discrimination (TID), which requires judging whether a test interval is longer (or shorter) than a standard interval (Fig. 1), cannot transfer from audition to vision^10^, inconsistent with the prediction of modality-unspecific time representation. Later studies found unidirectional transfer effects, in that TID learning only transfers (probably partially, see our data below) from audition to vision, but not vice versa^8,9^. The asymmetric learning transfer may be a result of dominant auditory temporal processing. The latter may also be responsible for time coding of other senses due to its high precision, which may not benefit from training-improved visual time processing that is still too coarse to be useful^8,9,19,20^. Nevertheless, this interpretation still implies modality-specific time representation.

The goal of this study is to demonstrate mutual and complete transfer of visual and auditory TID learning, so as to prove a supramodal subsecond time representation. Our previous perceptual learning studies have shown that various forms of specificities are not necessarily innate properties of perceptual learning as commonly believed, and can be eliminated with a double-training procedure^21–23^. In contrast to conventional training in which only the task of interest is practiced, double training consists of two training tasks. The primary training task in the current context would be TID in one sense (e.g., audition), and the secondary training task would be a functionally orthogonal one, such as contrast discrimination, in a new sense (e.g., vision). Here in the contrast discrimination task, the two Gabor gratings in a two-alternative forced-choice trial would mostly have near-threshold contrast differences and be presented at the same temporal interval as in the primary task, so that the participants would receive exposure to the temporal interval passively, but their attention is directed to near-threshold contrast discrimination to prevent potential temporal learning with the secondary task. The secondary task thus may activate sensory neurons representing the temporal interval in the new sense, so that the potential supramodal TID learning could functionally connect to temporal inputs from the new sense to improve TID performance. Double training has successfully enabled learning transfer of various visual discrimination tasks to untrained retinal location, orientation, motion direction, etc.^21,22,24–26^. It also succeeded in transferring auditory^27^ and visuomotor learning^28,29^.

Most relevant to the current study is our recent report that perceptual learning of tactile orientation discrimination can transfer completely to visual orientation discrimination after double training, even if no transfer was evident with conventional single training^30^. These results are interpreted as evidence for a supramodal representation of stimulus orientation. Moreover, since the tactile orientation threshold is about three times as high as the visual orientation threshold, learning transfer is possible only if the supramodal representation is abstract and conceptual, independent of the original modality precision of sensory inputs^30,31^. Following the same reasoning, here we hypothesized that if perceptual learning of auditory and visual TID, which also differ in precision, could transfer mutually and completely with double training, we would also have evidence for a supramodal representation of subsecond time at a conceptual level.

## Results

### Baselines: asymmetric learning transfer between auditory and visual TID with conventional single training

We first measured the cross-modal transfer of TID learning between audition and vision with conventional single training, which established baselines for later double training experiments. One group of participants (*N* = 7) practiced auditory TID (auditory single-training group), and a second group (*N* = 9) practiced visual TID (visual single-training group), both with the 100-ms standard interval.

**Figure 2:**
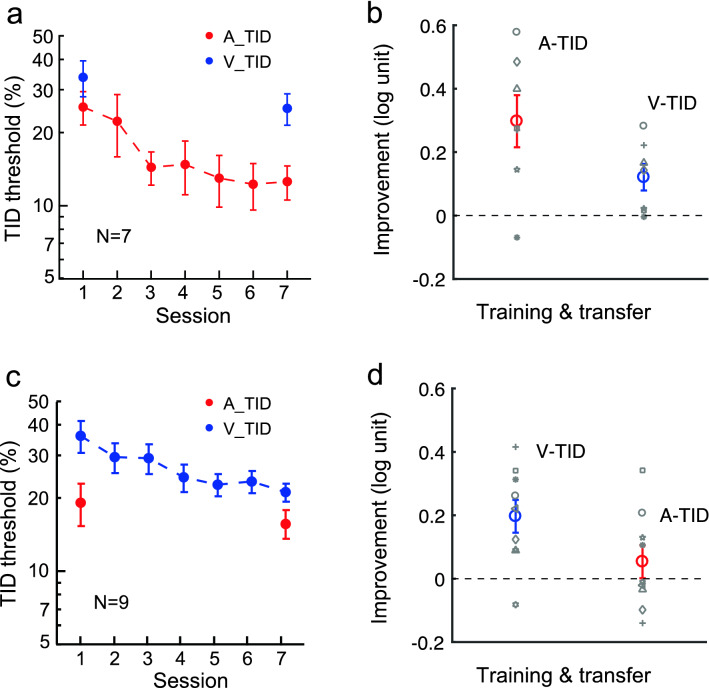
Baselines: Asymmetric audition-to-vision transfer in TID learning with single training. (**a**) The mean auditory temporal interval discrimination (A_TID) learning curve and pre- and post-training visual TID (V_TID) thresholds. (**b**) The mean and individual performance improvements with trained auditory TID and untrained visual TID. (**c**) The mean visual TID learning curve and the pre- and post-training auditory TID thresholds. (**d**) The mean and individual performance improvements with trained visual TID and untrained auditory TID. Here and in later figures, error bars indicate ± 1 standard error of the mean.

For the auditory single-training group, training reduced auditory TID threshold by 0.30 ± 0.08 log units (t_6_ = 3.63, *p* = 0.011, Cohen’s d = 1.37). The same training also improved visual TID at the same 100-ms interval, reducing visual TID threshold by 0.12 ± 0.04 log units (t_6_ = 3.87, *p* = 0.029, Cohen’s d = 1.08) (Fig. 2a, b). However, for the visual single-training group, although training improved visual TID by 0.20 ± 0.05 log units (t_8_ = 3.81, *p* = 0.005, Cohen’s d = 1.27), the learning did not transfer to auditory TID at the same interval (by 0.05 ± 0.05 log units; t_8_ = 1.04, *p* = 0.33, Cohen’s d = 0.35) (Fig. 2c, d). These results thus confirmed previous reports of asymmetric audition-to-vision transfer of TID learning with conventional single training^8,9^. Here the visual TID improvement through auditory TID training (V_TID in Fig. 2b) was about 60% of that through direct visual TID training (V_TID in Fig. 2d), suggesting that auditory TID training might have not maximized the visual TID performance in these observers. In other words, the audition-to-vision learning transfer was partial.

### Double training: complete vision-to-audition transfer of TID learning

**Figure 3:**
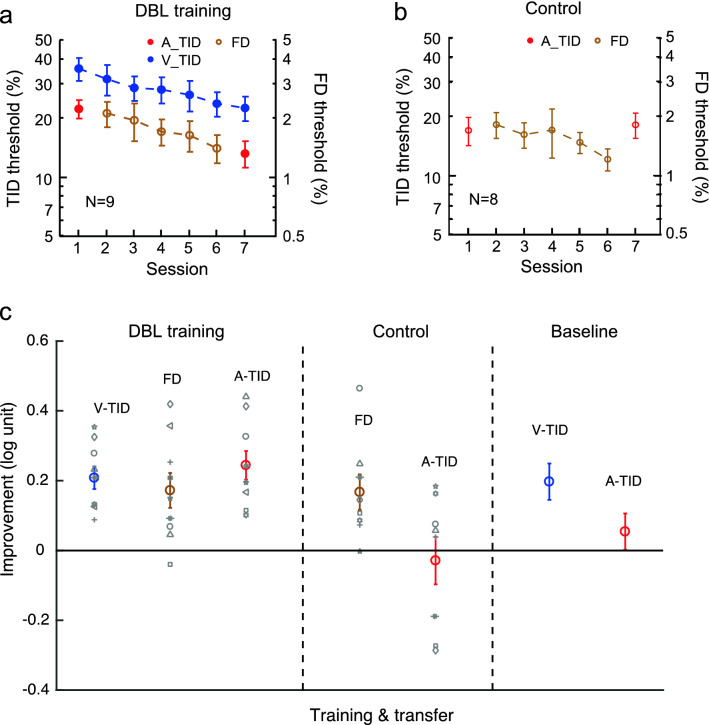
The cross-modal transfer of TID learning from vision to audition with double training. (**a**) Double training: The mean learning curves of visual TID (Sessions 1–7) and tone frequency discrimination (FD) (Sessions 2–6). Auditory TID was tested pre- and post-double training (Sessions 1 & 7). (**b**) Control: The impact of tone frequency discrimination training alone on auditory TID threshold. (**c**) The mean and individual improvements of visual and auditory TID thresholds and FD threshold in double training and control conditions, as well as the mean improvements of visual and auditory TID thresholds in the earlier single visual TID training condition (replotted from Fig. 2d).

Next, we examined whether visual TID learning could transfer to auditory TID with double training. Nine participants practiced visual TID at a 100-ms interval. They also received exposure to the auditory 100-ms interval by practicing an orthogonal tone frequency discrimination task at the same interval. This double training improved visual TID by 0.21 ± 0.03 log units (t_8_ = 6.54, *p* < 0.001, Cohen’s d = 2.18) and tone frequency discrimination by 0.17 ± 0.05 log units (t_8_ = 3.44, *p* = 0.009, Cohen’s d = 1.15) (Fig. 3a, c). Importantly, auditory TID at the same interval also showed an improvement of 0.24 ± 0.04 log units (t_8_ = 5.92, *p* < 0.001, Cohen’s d = 1.97) (Fig. 3c), which was not significantly different from the 0.29 log-unit improvement with direct auditory TID training in the auditory single-training group (Fig. 2b) (t_14_ = 0.63, *p* = 0.54, Cohen’s d = 0.31). Therefore, auditory TID appeared to have maximized after visual TID training and tone frequency discrimination training were coupled in double training, even if it was unaffected by visual TID training alone (Fig. 2c, d).

To exclude the possibility that the auditory TID improvement was simply a result of tone frequency discrimination training, we had a control group (*N* = 8) only practice tone frequency discrimination at a 100-ms interval. The practice improved tone frequency discrimination by 0.17 ± 0.05 log units (t_7_ = 3.27, *p* = 0.014, Cohen’s d = 1.16), but it failed to improve auditory TID at the same interval (by − 0.03 ± 0.07 log units; t_7_ =  − 0.43, p = 0.68, Cohen’s d =  − 0.15, Fig. 3b, c). Taken together, the double training results and control data suggested that double training enabled full learning transfer from visual TID to auditory TID, in spite of the insignificant transfer in the single-training condition (Fig. 2c, d).

To reduce Type-I errors in our data analysis, a between-subject ANOVA compared auditory TID improvements among the three training conditions, i.e. single visual TID training, current double training, and tone frequency discrimination training. The ANOVA outputs suggested a significant main effect of training condition (F_2, 24_ = 7.70, *p* = 0.003, η^2^ = 0.39). Further contrast analysis showed that the auditory TID improvement after double training was significantly higher than the improvement after single visual TID training (t_24_ = 2.60, *p* = 0.016) and the improvement after tone frequency discrimination training (t_26_ = 2.69, *p* = 0.012).

### Double training: complete audition-to-vision transfer of TID learning

**Figure 4:**
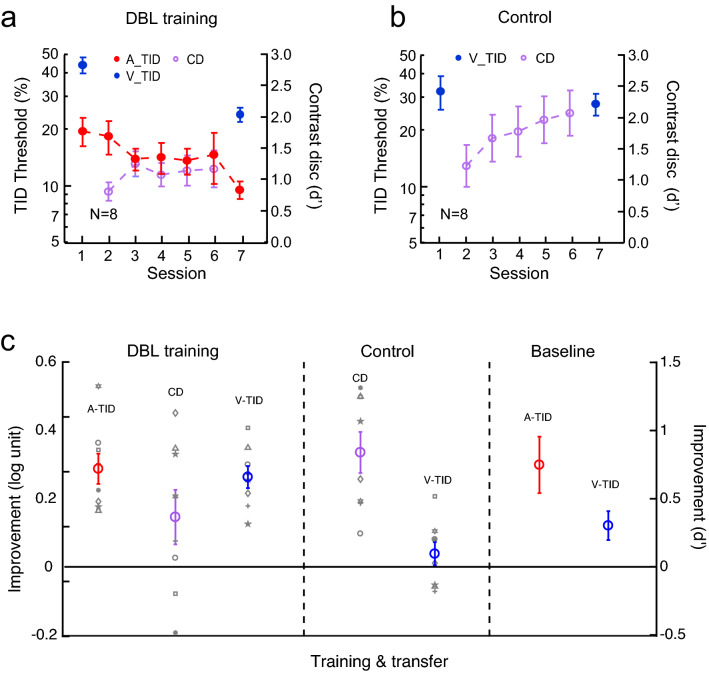
The cross-modal transfer of TID training from audition to vision with double training. (**a**) Double training: The mean learning curves of auditory TID (Sessions 1–7) and visual contrast discrimination (CD) (Sessions 2–6) at a 100-ms interval. Visual TID at the same interval was tested pre- and post-double training (Sessions 1 & 7). (**b**) Control: The impact of visual contrast discrimination training alone on visual TID threshold at the same interval. (**c**) The mean and individual improvements of auditory and visual TID thresholds and visual contrast discrimination threshold in double training and control conditions, as well as the mean improvements of auditory and visual TID thresholds in the earlier single A_TID training condition (replotted from Fig. 2b).

Earlier we suggested that visual TID improvement after auditory TID training was approximately 60% of that after direct visual TID training (Fig. 2b, d). Here we examined whether double training could lead to complete audition-to-vision TID learning transfer. Eight new participants practiced auditory TID and visual contrast discrimination, both at a 100-ms interval, in alternating blocks of trials in the same training sessions. Training improved auditory TID by 0.29 ± 0.04 log units (t_7_ = 6.55, *p* < 0.001, Cohen’s d = 2.32) and visual contrast discrimination by 0.37 ± 0.20 in d’ (t_7_ = 2.06, *p* = 0.058, Cohen’s d = 0.73), as well as visual TID at the same interval by 0.26 ± 0.03 log units (t_7_ = 8.21, *p* < 0.001, Cohen’s d = 2.90) (Fig. 4a, c). The visual TID improvement did not differ significantly from the 0.20 log-unit improvement through direct visual TID training (Fig. 2c) (t_15_ = 1.03, *p* = 0.31, Cohen’s d = 0.50), suggesting that the visual TID performance had maximized after double training.

Again, a control group of participants (*N* = 8) practiced visual contrast discrimination only, which improved contrast threshold by 0.84 ± 0.15 in d’ (t_7_ = 4.74, *p* < 0.001, Cohen’s d = 1.68). But this practice had no significant impact on visual TID at the same 100-ms interval (by 0.04 ± 0.03 log units; t_7_ = 1.13, *p* = 0.30, Cohen’s d = 0.30, Fig. 4b, c), excluding the possibility that contrast discrimination training per se was responsible for above visual TID learning after double training. Here the visual pretraining threshold (V_TID) appeared to be lower than that with the double training group, which was mainly due to one participant who showed very low pre-training threshold at 11.8%. The pre-training V-TID thresholds were not significantly different from each other (p = 0.146, two-tailed independent t-test).

To reduce Type-I errors in data analysis, a between-subject ANOVA compared visual TID improvements after single auditory TID training, current double training, and contrast discrimination training. The ANOVA outputs indicated a significant main effect of training condition (F_2, 21_ = 10.31, *p* < 0.001, η^2^ = 0.51). Further contrast analysis indicated that the TID improvement after double training was significantly higher than the improvement after single auditory TID training (t_20_ = 2.74, *p* = 0.013) and from the improvement after contrast discrimination training (t_20_ = 3.23, *p* = 0.004), confirming that double training induced more audition-to-vision TID learning transfer than auditory TID training alone, and that the double training effect could not be accounted for by visual contrast discrimination training.

## Discussion

In this study we demonstrate mutual and complete cross-modal transfer of auditory and visual TID learning with double training, regardless of the difference in timing precisions (thresholds) between two senses, as well as the asymmetric audition-to-vision transfer of TID learning with conventional (single) training. These data thus provide direct support for a supramodal representation of subsecond time that can be improved through perceptual learning. Our results are consistent with previous reports which have also suggested supramodal subsecond time representation, on the basis of computer simulation^12^, structure equation modeling of experimental data^14^, and more direct crossmodal interference of duration judgments^13^ and EEG data^11^. Evidence for a supramodal representation of subsecond time is in line with hypotheses of a dedicated central clock^1–3^ that participates in subsecond time perception, although these hypotheses do not necessarily contradict the roles of distributed timing mechanisms^14^.

The auditory and visual subsecond time information differs in not only modality origin, but also precision (the auditory TID threshold is approximately half the visual TID threshold, Figs. 2, 3, 4). Therefore, the double training results suggest complete cross-modal as well as cross-precision TID learning transfer. The cross-precision learning transfer would suggest that the time inputs from different modalities are represented equally at a supramodal level, which could be achieved through abstraction or standardization of the time inputs by their respective precisions (i.e., standard deviations). It is in this sense that we interpret the cross-modal TID learning transfer data as indications of not only supramodal, but also conceptual, representation of subsecond time. It is worth mentioning that the cross-modal TID learning transfer may not be a result of training-improved general decision-making strategies, as TID learning is task specific (Figs. 3b, 4b). Perceptual learning remains task specific even with double training^32^.

The cross-modal TID learning transfer results suggest that the conceptual and supramodal representation of subsecond time can be improved through TID training in one sense, and subsequently connects to a new sense to improve TID performance with double training. Consistent with this argument, Nagarajan, et al.^33^ reported that coarser somatosensory TID learning transfers to finer auditory TID, which is only possible when somatosensory and auditory temporal inputs are standardized to be equal at a conceptual level. The supramodal and conceptual representation of subsecond time is different from simple cross-modal time coding that still integrates time cues from different senses on the basis of their respective precisions, which would predict asymmetric audition-to-vision learning transfer^9^. Furthermore, it is different from a supramodal internal reference in the memory formed through repetitive practice for a specific interval^17,34^, as the latter would also have different precisions depending on whether an auditory or visual interval is practiced, which again would predict asymmetric learning transfer^8^.

Why does initially modality specific TID learning become transferrable after double training? Although we do not have direct evidence for an explanation, hints may come from our visual perceptual learning studies. We once reported that location and orientation specificity associated with Vernier learning can be eliminated if an untrained transfer location or orientation is activated with bottom-up stimulation and/or top-down attention^23^. Moreover, an ERP study^35^ revealed that learning transfer to an untrained location is associated with N1-P1 changes. These results indicate that the specificity of visual perceptual learning may be related to missing or weak functional connections from high-level learning to sensory inputs from the untrained retinal location or representing the untrained orientation, and double training may establish or strengthen these connections to enable learning transfer. Similarly, the current modality specificity and transfer of TID learning may be also related to the strength of functional connections between high-order TID learning and temporal inputs from an untrained modality, which varies as a result of single or double training.

Although distributed mechanisms have been overwhelmingly favored^3–5^, some accumulating evidence supports that both central and distributed timing mechanisms may contribute to subsecond time perception^11,14–16^. Our results are consistent with these hybrid accounts. The asymmetric transfer of TID learning can be seen as evidence for the involvement of separate distributed mechanisms since coarse visual TID learning has little direct impact on finer auditory TID performance. However, the cross-modal transfer of auditory and visual TID learning revealed with double training may also suggest additional engagement of a dedicated central clock. Therefore, both central and distributed timing mechanisms may contribute to subsecond time perception and its improvement through training.

## Methods

### Participants and apparatus

Data were collected from 49 college students (36 females, 20.9 ± 2.2 years old) who had normal or corrected-to-normal vision and normal hearing (pure-tone thresholds ≤ 20 dB hearing level across 0.5–6 kHz). They were inexperienced with visual psychophysical or psychoacoustic experiments and naïve to the purpose of the study. Informed consent was obtained from each participant prior to data collection. The study was approved by the Peking University IRB, and was carried out in accordance with the Code of Ethics of the World Medical Association (Declaration of Helsinki).

Experiments were run in an anechoic booth. The stimuli were generated with a Matlab-based Psychtoolbox-3^36^. Auditory stimuli were diotic, presented by a pair of Sennheiser HD-499 headphones. Visual stimuli were presented on a 19-inch Sony G420 CRT monitor with a resolution of 800 pixel × 600 pixel and a refresh rate of 160 Hz. The luminance of the monitor was linearized by an 8-bit look-up table, with a mean luminance of 43.5 cd/m^2^. A chin-and-head rest stabilized the head of the observer.

### Stimuli and procedures

The auditory stimuli were two 15-ms tone pips separated by a 100 ms standard temporal interval (Fig. 1a). Each tone contained a 5-ms cosine ramp at each end, and was fixed at 1 kHz and 86 dB SPL. The visual stimuli were two 15-ms Gabor gratings, also separated by a 100 ms interval (Fig. 1b). Each Gabor had a fixed orientation (vertical), spatial frequency (1 cycle/deg), and contrast (100%). The length of the interval was the difference between the offset of the first stimulus and the onset of the second stimulus. We used 100 ms as the standard temporal interval because previous studies had shown clear evidence for significant TID learning and asymmetric audition-to-vision learning transfer at this interval^8^.

The TID threshold was measured with a method of constant stimuli. In each forced-choice trial, a visual fixation was first centered on the computer screen for 300 ms, then two pairs of stimuli, one with a standard interval (100 ms) and the other with a comparison interval (100 ms + Δt), were subsequently presented in random order with a 900-ms time gap. The participants pressed the left or right arrow to indicate whether the first or the second pair of stimuli had a longer interval. A happy or sad cartoon face was shown on the screen after each response to indicate a correct or wrong response. A blank screen was presented before the next trial for a random duration (500-1000 ms). The Δt was set at 6 levels for each condition (auditory TID: ± 20.1, ± 13.4, ± 6.7 ms; visual TID: ± 33.5, ± 20.1, ± 6.7 ms), and the intervals between stimulus levels were increased if necessary to ensure a sufficient range of correct rates. Each level was repeated 10 times in a block of 60 trials, for a total of 5 blocks.

The psychometric function was fitted with *P* = $\frac{1}{1+{e}^{\left(-k\right)*(\mathrm{\Delta t}-{\mathrm{\Delta t}}_{0})}}$, where *P* was the rate of reporting the comparison interval being longer at each Δt, k was the slope, and Δt_0_ was the point of subjective equivalence. The TID threshold was equal to half the interquartile range of the function: Threshold = (Δt_.75_ − Δt_.25_)/2.

The stimuli for tone frequency discrimination were the same as those for auditory temporal interval discrimination, except that the frequencies of two pairs of pips were changed while the temporal intervals were fixed at 100 ms. Two pairs of tone pips, one pair at a standard frequency of 1 kHz and the other at a higher comparison frequency (1 kHz + Δf), were presented subsequently in a random order in each trial. The participants pressed the left or right arrow to indicate whether the first or second pair of tone pips had a higher frequency. A happy or sad cartoon face was provided as feedback.

The tone frequency discrimination threshold was measured with a temporal 2AFC staircase procedure. The starting frequency difference (Δf) between the standard and comparison stimuli was 50%, which decreased by a factor of 2 after every correct response until the first incorrect response. Then the Δf was varied by a factor of 1.414 following a 3-down-1-up staircase rule for a 79% correct rate. Each staircase ended after 60 trials. The threshold was calculated as the mean of the last 40 trials.

The stimuli used for visual contrast discrimination were the same as those for visual temporal interval discrimination, except that the Gabor contrast was varied while the interval was fixed (100 ms). Only one pair of Gabors was presented in each trial. In 80% of the trials, the two Gabors had identical contrast, which randomized from 0.15 to 1. In the remaining 20% trials, the contrasts of two Gabors differed by 50%. The participants judged whether two Gabors had identical contrast. A happy or sad cartoon face was provided as feedback. The d’ value was calculated to measure the contrast discrimination performance.

Each experiment consisted of a pre-training session, five training sessions, and a post-training session on separate days. The experiment was completed within 7–13 days, with inter-session gaps of no more than 2 days. Each single-training session consisted of 16 blocks of trials and lasted for approximately 1.5 h. Each double-training session consisted of 10 blocks of trials for the primary task and 10 blocks of trials for the secondary task in an alternating order, and lasted for approximately 2 h.

### Sample size

The sample size was decided on the basis of a previous TID learning study that used similar stimuli (100 ms–1 kHz condition in Fig. 4, ref.^18^). In our study, learning and transfer involved comparisons between pre- to post-training thresholds in all experiments. To achieve 80% power at *p* = 0.05, for a similar effect size of Cohen’s d = 1.34 in ref.^18^ when comparing pre- and post-training thresholds, a sample size of 7 would be required. We used a sample size of 9 for each experiment, with consideration of potential dropout of participants.

### Data analysis

The TID thresholds were log-transformed to achieve normal distributions (Shapiro–Wilk test before log-transformation: *p* < 0.001 for auditory and visual TID thresholds; Shapiro–Wilk test after log-transformation: *p* = 0.28 and 0.60 for corresponding TID thresholds). The amount of TID learning or transfer was then measured by the difference of pre- and post-training thresholds in log unit. Data were analyzed with JASP 0.14.1. A two-tailed one-sampled t-test was performed to examine whether a learning or transfer effect was different from 0, and a between-subject ANOVA with Bonferroni's correction was performed for multiple comparisons.

## Data Availability

Data are available at https://github.com/visionplusplu/ModalityLearning.
